# Low asparagine wheat: Europe’s first field trial of genome edited wheat amid rapidly changing regulations on acrylamide in food and genome editing of crops

**DOI:** 10.1270/jsbbs.23058

**Published:** 2024-03-20

**Authors:** Navneet Kaur, Natasha Brock, Sarah Raffan, Nigel G. Halford

**Affiliations:** 1 Rothamsted Research, Harpenden AL5 2JQ, United Kingdom; 2 The Salk Institute for Biological Studies, 10010 N Torrey Pines Rd, La Jolla, CA 92037-1002, United States of America

**Keywords:** acrylamide, asparagine, CRISPR, food safety, precision breeding organisms, processing contaminants, wheat

## Abstract

We review the undertaking of a field trial of low asparagine wheat lines in which the asparagine synthetase gene, *TaASN2*, has been knocked out using CRISPR/Cas9. The field trial was undertaken in 2021–2022 and represented the first field release of genome edited wheat in Europe. The year of the field trial and the period since have seen rapid changes in the regulations covering both the field release and commercialisation of genome edited crops in the UK. These historic developments are reviewed in detail. Free asparagine is the precursor for acrylamide formation during high-temperature cooking and processing of grains, tubers, storage roots, beans and other crop products. Consequently, work on reducing the free asparagine concentration of wheat and other cereal grains, as well as the tubers, beans and storage roots of other crops, is driven by the need for food businesses to comply with current and potential future regulations on acrylamide content of foods. The topic illustrates how strategic and applied crop research is driven by regulations and also needs a supportive regulatory environment in which to thrive.

## Introduction

### The problem of acrylamide in food and the need for low asparagine wheat

Acrylamide (C_3_H_5_NO) is a white, odourless, crystalline, water-soluble solid, familiar to anyone who works in a biochemistry/molecular biology laboratory, where it is used in its polymeric form to make gels used in electrophoresis. In its monomeric form, it is regarded as a hazardous chemical, classified as a Group 2a carcinogen (probably carcinogenic to humans) by the International Agency for Research on Cancer (IARC) (IARC 1994), and as ‘reasonably anticipated to be a human carcinogen’ by the USA’s National Toxicology Program (NTP 2021). The European Food Safety Authority (EFSA)’s Scientific Panel on Contaminants in the Food Chain (CONTAM) considers acrylamide to be neurotoxic, mutagenic, genotoxic and carcinogenic, with negative effects on development and fertility (EFSA Panel on Contaminants in the Food Chain (CONTAM) 2015).

Not surprisingly, therefore, the discovery of acrylamide in food in 2002 (Tareke *et al.* 2002) caused something of a seismic shock in the food industry. Since that discovery, the European Union (EU) has been screening food products for the presence of acrylamide and imposing increasingly difficult ‘risk management’ measures on food businesses. The current regulations were introduced in 2017 (Regulation (EU) 2017/2158 (European Commission 2017)), establishing Benchmark Levels for the presence of acrylamide in different food types and setting out compulsory mitigation methods that all food businesses have to adopt. Benchmark Levels are described as ‘performance indicators’ for the mitigation measures that food businesses use. The current Benchmark Levels include 50 μg kg^–1^ (parts per billion) for soft bread, 350 μg kg^–1^ for biscuits and crispbreads, and 300 μg kg^–1^ for breakfast cereals. In Japan, the Ministry of Agriculture, Fisheries and Food has implemented an acrylamide monitoring programme and issued a code of practice for the reduction of acrylamide in food (MAFF 2009).

Compliance with Regulation (EU) 2017/2158 is already a difficult issue for the EU food industry. Recent studies conducted in Spain, for example, found that 30% of biscuits and 15% of breakfast cereals contained acrylamide above the Benchmark Level (Mesías *et al.* 2019a, 2019b). However, the situation looks set to get much more problematic with the imminent lowering of Benchmark Levels and imposition of Maximum Levels (above which it would be illegal to sell a product) for some foods, including all of the major cereal products (European Commission 2022). It would, of course, be illegal to sell food containing acrylamide above the Maximum Level. The Maximum Levels that are reportedly being discussed include 75 μg kg^–1^ for soft bread, 500 μg kg^–1^ for biscuits, 400 μg kg^–1^ for crispbreads, 500 μg kg^–1^ for wholegrain breakfast cereals and 400 μg kg^–1^ for other wheat-based breakfast cereals. These may not be the levels that are eventually imposed, but if they are, we would expect there to be some failures, resulting in severe consequences for food businesses, including product recalls.

Regulation (EU) 2017/2158 rolled over into United Kingdom (UK) law when the UK left the EU in 2020. The UK does not have to follow the EU’s lead in setting Maximum Levels for acrylamide, but at the time of writing there is no indication of whether or not it will. UK food businesses and their suppliers, therefore, face uncertainty over whether the EU (the UK’s largest trading partner) is about to impose Maximum Levels for acrylamide, what those levels will be, and whether Maximum Levels will also be applied in the UK.

Acrylamide is present in foods prepared from grains, tubers, storage roots and beans, including coffee. It is not present in the crop itself, but forms during cooking and processing; i.e., it is a processing contaminant. The major route for its formation is within the Maillard reaction, a complex series of non-enzymic chemical reactions taking place between free (soluble, non-protein) amino acids and reducing sugars (reviewed by Kaur and Halford 2023). The Maillard reaction can occur at low temperatures, but is greatly accelerated by heat, and it is at temperatures >120°C that it becomes relevant to food processing. These temperatures are, of course, associated with frying, baking, roasting and toasting. The reaction is multistep, with free amino acids participating in the early and later stages. It generates multiple products, many of which contribute to the colours, flavours and aromas that are associated with fried, baked, roasted and toasted foods, and are demanded by consumers. However, when free asparagine participates in the later stages of the reaction, acrylamide forms (Mottram *et al.* 2002, Stadler *et al.* 2002).

There are other routes for acrylamide formation from free asparagine. One involves 3-aminopropionamide (3-APA) as a transient intermediate, with deamination of 3-APA resulting in acrylamide formation even in the absence of reducing sugars (Granvogl and Schieberle 2006); another, a direct reaction between free asparagine and reducing sugars (Parker *et al.* 2012). Other compounds have also been proposed to act as precursors for acrylamide, including acrolein, acrylic acid and wheat gluten (Claus *et al.* 2006, Yasuhara *et al.* 2003). However, acrylamide formation in heated wheat and rye flour correlates closely with free asparagine concentration (reviewed by Raffan and Halford 2019).

Food manufacturers began working to reduce the levels of acrylamide in their products almost as soon as the problem was discovered, and the methods they have developed have been compiled in a ‘Toolbox’ produced by FoodDrinkEurope (FoodDrinkEurope 2019). However, analysis of data on potato crisps (US chips) produced in Europe shows that the big gains in reducing acrylamide content were made in the first decade after acrylamide was discovered in food, with the levels plateauing after 2011 (Powers *et al.* 2021). There is no reason to expect the cereals sector to be any different.

One impediment to progress in reducing acrylamide levels in some products is that mitigation methods for acrylamide risk leaving manufacturers with insipid products. That could be addressed by providing manufacturers with lower asparagine grains that could be used to produce foods that comply with regulations while retaining the characteristics that consumers demand. Conversely, it is essential that grains with very high concentrations of free asparagine do not enter a manufacturer’s production system because they could cause a spike in acrylamide in the product. This would be catastrophic if Maximum Levels for acrylamide were in place. Agronomy plays a big part here, notably in ensuring that wheat is supplied with adequate sulphur and is protected from pathogen infection (reviewed by Kaur and Halford 2023). Nevertheless, food businesses would benefit greatly from the availability of wheat grain that was intrinsically low in free asparagine concentration, and less prone to accumulating high concentrations of free asparagine in response to environmental factors.

## Engaging breeders and providing tools/resources for producing low asparagine wheat

Considerable efforts have been made around the world to provide wheat breeders with the resources and knowledge to produce wheat varieties with reduced free asparagine concentration in the grain, including varietal comparisons over multiple field trials and the identification of quantitative trait loci (QTL) that control free asparagine accumulation (reviewed by Kaur and Halford 2023). However, while breeders have supported some of this work, to our knowledge they have not adopted reduced free asparagine concentration as a breeding target anywhere in the world. Consequently, it is fair to say that absolutely no progress has been made in reducing the free asparagine content of commercial wheat varieties, 21 years after the discovery of acrylamide in food. Clearly, engaging breeders on this issue will be key to driving improvement.

On the face of it, there is considerable natural variation in free asparagine to work with (see, for example, Curtis *et al.* 2018, Tafuri *et al.* 2023). The interaction of genetics with environmental and crop management factors (G × E) undoubtedly makes it difficult to exploit this variation, but breeders deal with G × E interactions on other traits, so that should not preclude the possibility of reducing free asparagine concentration by breeding. It is also true that the QTL that have been identified for free asparagine concentration have been weak and inconsistent (reviewed by Kaur and Halford 2023). Oddy *et al.* (2023), for example, identified a QTL for free asparagine concentration by analysing a doubled haploid mapping population produced from a cross between varieties Claire and Robigus, two varieties that have been extensively used as parent lines in soft wheat breeding in the UK. The QTL was located on chromosome 4B, only 60 Mbp away from a QTL reported in a previous study (Peng *et al.* 2018), to our knowledge the first time that two studies had identified QTL that might overlap. However, while this QTL was responsible for 14.8% of the variance in a 2018–2019 field trial, it accounted for only 2.6% of the variance in the previous year’s field trial.

Despite the lack of strong QTL for the trait, there is a genetic variant that should be exploitable to reduce free asparagine concentration in the grain. Wheat has five asparagine synthetase genes: *TaASN1*, *TaASN2*, *TaASN3.1*, *TaASN3.2* and *TaASN4* (Raffan and Halford 2021). This gene family structure is unique to the Triticeae and, importantly, one of those genes, *TaASN2*, is expressed grain-specifically and is by far the most highly expressed *ASN* gene in the grain by mid-development (Gao *et al.* 2016). Analysis of wheat genome data showed that variety Chinese Spring lacked a *TaASN2* gene on the B genome (*TaASN-B2*) as a result of a natural deletion of almost 13 kb (Xu *et al.* 2018). A study by Oddy *et al.* (2021) showed that the deletion encompassing the *TaASN-B2* gene was present in most but not all cultivated varieties, as well as some wild emmer wheat genotypes. Furthermore, there was a trend for free asparagine concentrations in field-produced grain to be lower in varieties lacking *TaASN-B2*, as long as the wheat was grown with an adequate sulphur supply.

Overall, these studies suggest that there may be limited scope for reducing free asparagine concentrations by conventional breeding. Exploiting the absence of the *TaASN-B2* gene in some varieties looks to be the most promising strategy. The fact that most varieties already lack the gene, across all milling types, limits the usefulness of the variant, but breeders could already be removing genotypes that still have a *TaASN-B2* gene from their breeding programmes for a relatively quick and easy gain. It is extremely disappointing that breeders are failing to do this and, in our view, additional motivation is required. This could be in the form of a requirement to acquire data on free asparagine content in the grain during variety development and make the information available on variety launch.

## Production of low asparagine wheat by CRISPR/Cas9

The advent of genome or gene editing (GE) using the Clustered Regularly Interspaced Short Palindromic Repeats (CRISPR) system along with a CRISPR-associated (Cas) endonuclease raised the possibility of knocking out one or more of the asparagine synthetase genes of wheat. Indeed, the wheat *ASN* gene family seemed particularly well suited to the system, with single genes per genome and one obvious target in *TaASN2*, which is expressed grain-specifically. The hypothesis was that knocking this gene out would reduce the accumulation of free asparagine in the grain while the remaining genes would provide sufficient asparagine for protein synthesis. This was based on the assumption that the relatively high concentrations of free asparagine compared with other free amino acids in wheat grain (Curtis *et al.* 2009) indicated that the free asparagine was being used for purposes in addition to synthesising proteins, such as acting as a nitrogen storage and transport molecule.

CRISPR/Cas9 together with four guide RNAs (gRNAs) were used to knock out the *TaASN2* gene of wheat cv. Cadenza (Raffan *et al.* 2021). Cadenza can be drilled in the autumn or spring and was a commercial wheat variety in the UK in the 1990s and 2000s. It continues to be used for research because it is relatively easy to transform. The four gRNA-encoding DNAs were incorporated into a single, polycistronic gene, separated by tRNA sequences, based on the system developed by Xie *et al.* (2015). This system exploits the endogenous tRNA-processing system to generate multiple gRNAs from a single transgene, enabling multiplex editing. It was chosen because of the recalcitrance of wheat to transformation, to increase the chances of an effective gRNA being introduced in one transformation event. The construct was assembled downstream of a rice small nucleolar RNA U3 (*U3sno*) gene promoter. The *Cas9* gene had been codon-optimised for wheat and was downstream of a maize *Ubi1* promoter plus first intron. A selectable marker gene (*bar*) encoding phosphinothrycin acetyl transferase (PAT), also under the control of a maize *Ubi1* gene promoter and first intron, was present in a third plasmid. The use of three separate plasmids undoubtedly made it more difficult to segregate the transgenes away once the editing was completed and, if we were to repeat the experiment, we would attempt to produce a single plasmid carrying all three transgenes. The three plasmids were introduced into scutella of wheat cv. Cadenza embryos by particle bombardment.

**Fig. 1.**
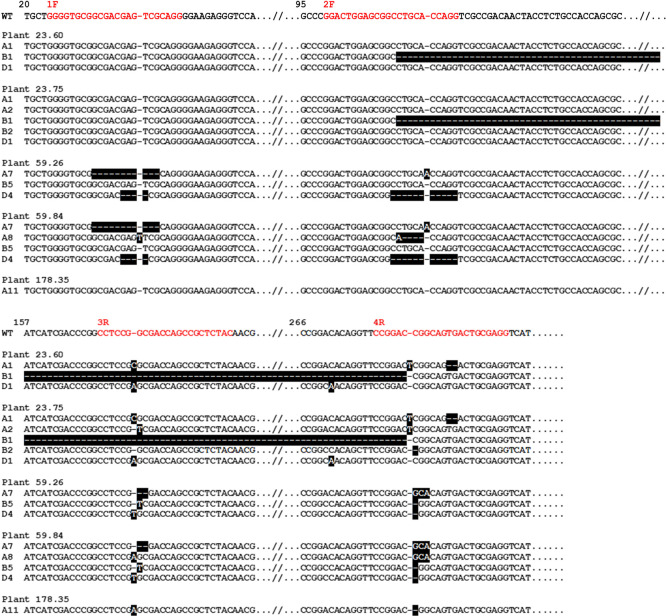
Nucleotide sequences showing the edited *TaASN2* alleles present in lines used in the field trial. The A genome wildtype sequence is shown at the top, with the gRNA binding sites in red and labelled (1F, 2F, 3R and 4R). The top and bottom panels show continuous nucleotide sequences for the alleles in the plants from which the lines were derived. Mutations induced by the editing process are highlighted in black. SNPs present in the wildtype sequences of the genes from genomes B and D are not highlighted. The different alleles present are numbered. If more than one allele is shown for one of the genomes of a plant it means that the plant was heterozygous, with two different edited alleles for that gene. For example, plant 23.75 was heterozygous for both the A and B genomes, with alleles A1 and A2 as well as B1 and B2. Adapted from Raffan *et al.* (2021).

T1 plants derived from 11 of 14 T0 plants were shown to carry mutations induced by the editing process, representing a very high mutation rate, possibly due to the use of the polycistronic gRNA gene. Next generation sequencing (NGS) was used to characterise the mutations that had been introduced: most mutations were deletions (up to 173 base pairs), but there were also some single base pair insertions and substitutions. The single base pair insertions presumably resulted from the propensity of Cas9 to leave a single nucleotide 5′ overhang at the cleavage site, which is filled in before the DNA strands are re-ligated (Lemos *et al.* 2018). Lines derived from three of the edited plants (23, 59 and 178) were selected for the field trial, based on the NGS analysis. Plants 23 and 59 carried mutations in all six *TaASN2* alleles (i.e., both alleles of each genome, A, B and D), while plant 178 was edited only in the A genome alleles. Individual plants within the T3 generation derived from these plants showed some allelic variation (Fig. 1). Of the two plants of Line 23 that were chosen to produce seed for the field trial, for example, 23.60 was homozygous for alleles A1, B1 and D1 as shown in Fig. 1, while 23.75 was biallelic for alleles A1 and A2, biallelic for B1 and B2, and homozygous for D1. For line 59, 59.26 was homozygous for alleles A7, whereas 59.84 was biallelic for alleles A7 and A8, and both plants were homozygous for alleles B5 and D4 (Fig. 1). A single plant, 178.35, was chosen for Line 178: This plant was homozygous for allele A11 (Fig. 1) and had wild-type alleles for the B and D genomes. The proteins encoded by the edited alleles are all extensively truncated, with the exception of that encoded by allele A7, which is almost full-length but lacks the key glutamine binding domain.

Since the use of CRISPR/Cas9 with wheat is still relatively new, it is worth considering some of the lessons learned in the production of the *TaASN2* edited lines. The first of these was the problem for a hexaploid species of identifying plants carrying edited genes. Our initial attempts to use PCR to confirm that editing had taken place were inconclusive and so we resorted to NGS. The size and depth of the NGS data required to characterise the mutations induced by the editing process was challenging, and this was complicated by the reappearance of wildtype alleles in the T2 generation of some lines when none had been detected in the T1 generation. We assume this arose because some of the mutations that we were detecting were somatic only. As reported by Zhang *et al.* (2019), editing continued beyond the T0 generation in some of the lines, further complicating the characterisation of the mutations that had occurred. We conclude that several generations may be required and possibly the segregating away of the *Cas9* and/or gRNA genes to achieve stability.

Nevertheless, the study achieved its aim of producing wheat plants with a step reduction in free asparagine concentration in the grain. The concentrations of free asparagine in the grain of Line 178 plants grown under glass were 56% and 68% of controls over two generations, while those of line 23 were 43% and 57% of controls and Line 59 were 9% and 48% of controls (Raffan *et al.* 2021). The reduced free asparagine concentration was associated with increases in free glutamine, glutamate and aspartate. In other words, the partitioning between free asparagine, glutamine, aspartate and glutamate was altered, reflecting the fact that asparagine synthetase acts by transferring an amino group from glutamine to aspartate, producing asparagine and glutamate.

The next phase of the project was to undertake field trials to determine whether the low asparagine phenotype was maintained under field conditions and assess the performance of the lines with respect to emergence, yield, thousand grain weight (TGW), and composition. Seed (T4 generation) from the selected plants was sown under glass to produce enough seed for the field trial; hence, the seed used for the field trial were the T5 generation. The process of segregating the transgenes away in the lines used for the field trial was still ongoing: plants 178.35, 23.75 and 59.84 all lacked the *Cas9* gene, for example, but plants 23.60 and 59.26 were still positive for *Cas9*. This meant that the field trial was held under the European Union’s regulations on deliberate release of genetically modified (GM) organisms (GMOs), which rolled over into UK law when the UK left the EU in 2020 and to date have not been reformed. Consent to release the GMOs was granted by the UK government on 31/08/2021 (reference 21/R08/01).

Prior to planting of the field trial, one of the lines, Line 59, was found to carry a 36 bp deletion at the gRNA3 site in the related gene, *TaASN1*. *TaASN1* does contain a PAM sequence at this site, but it has two mismatches with gRNA3, so it was somewhat surprising to discover that editing had occurred. No editing had occurred at the other sites in *TaASN1*, or in any of the other *ASN* genes. This can be explained by the number of mismatches at the four potential gRNA binding sites in *TaASN1*, *TaASN3.1*, *TaASN3.2* and *TaASN4*, and the presence/absence of a PAM sequence, as shown in Table 1. Details on the edit in the *TaASN1* gene in lines 59.26 and 59.84 were not included in the application for consent to release a GMO, and under the rules operating at the time we were not able to include those lines in the field trial.

**Fig. 2.**
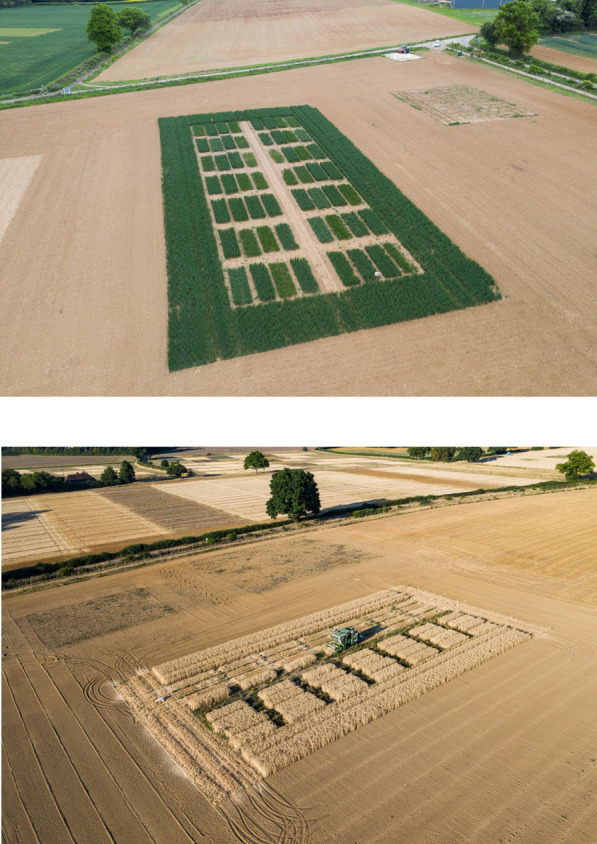
Drone pictures of the 2021–2022 field-trial, showing the layout of 56 plots of genome edited (Cadenza background) and TILLING mutant lines (Claire background) with respective controls, in a randomized block design. The plots were surrounded by a pollen barrier of Cadenza wheat and a 20m clear zone. The upper image shows the vegetative growth phase (May 2022) and the lower image the harvest (August 2022).

The field trial proceeded with Lines 178.35, 23.60 and 23.75 (Raffan *et al.* 2023). Also included were four AB genome nulls, referred to as TILLING lines 1–4, derived from a selected line of a mutant population produced by ethyl methanesulphonate treatment of wheat cv. Cadenza seeds (Rakszegi *et al.* 2010). The mutated *TaASN2-A2* gene from this line was backcrossed into the cv. Claire background to generate AB genome nulls. Claire was a popular biscuit, winter wheat variety in the UK in the 2000s–2010s and lacks a B genome *TaASN2* gene due to the ‘natural’ deletion described above (Oddy *et al.* 2021). The plants used in the field trial were of the BC2F3 generation. There were also control plots of cv. Cadenza, cv. Claire and a TILLING control that had come through the TILLING and backcrossing process but did not contain mutations in *TaASN2-A2*. The plots were drilled at a rate of 350 seeds per square metre, with rows 15 cm apart. Drone pictures taken of the trial in May 2022 and at harvest in August 2022 are presented in Fig. 2.

**Fig. 3.**
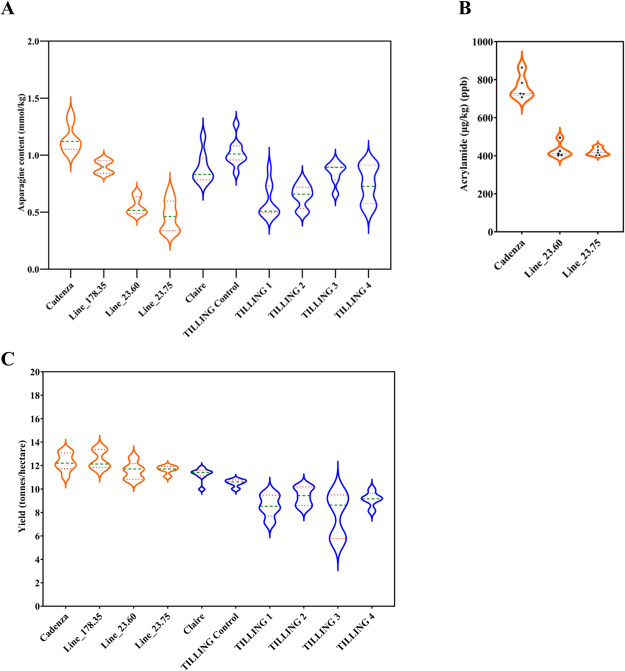
Violin plots representing free asparagine concentration, acrylamide formation and yield. A. Free asparagine content (mmol/kg) across genome-edited lines (in orange) and TILLING mutant lines (in blue), and their respective controls (Cadenza, Claire and TILLING control). B. Acrylamide content (μg/kg) in heated flour from genome-edited lines 23.60 and 23.85 compared to Cadenza. Green and dotted orange lines within the violin plots represent the median and upper/lower quartiles, respectively. C. Violin plots representing grain yield (tonnes/ha) across genome-edited (in orange) and TILLING mutants (in blue), and respective controls (Cadenza, Claire and TILLING control). Grain weight was taken for entire plots and yield calculated at 85% dry matter. Green and dotted orange lines within the violin plots represent the median and upper/lower quartiles, respectively. Plotted using data from Raffan *et al.* (2023).

The *TaASN2* null GE lines, 23.60 and 23.75, showed a significant reduction of approximately 50% in free asparagine concentration in the grain, compared with the Cadenza control (F_1,34.8_ = 74.95, *p* < 0.001), while the A genome null, Line 178.35, showed a reduction of 14% (Raffan *et al.* 2023) (Fig. 3A). The GE lines also showed significant increases in free glutamine (F_1,30.7_ = 102.49, *p* < 0.001). The situation was not so clear for the TILLING lines, which did show significant reductions of 20–40% in free asparagine compared with the TILLING control (F_1,35.7_ = 26.57, *p* < 0.001), but not when compared to Claire. This requires further investigation, but the reduction compared with the TILLING control was similar to that seen previously in A genome TILLING nulls in the Cadenza and Kronos backgrounds (Alarcón-Reverte *et al.* 2022).

Acrylamide was measured in wholemeal flour prepared from grains of lines 23.64 and 23.75, as well as the Cadenza control, after heating the flour for 20 minutes at 160°C. Acrylamide levels in the flour from the edited lines was 44–45% lower than in the Cadenza control (Fig. 3B) (*p* < 0.001), reflecting the decrease in free asparagine concentration (there was 427 and 421 μg kg^–1^ in flour from Lines 23.60 and 23.75, respectively, and 761 μg kg^–1^ in flour from Cadenza).

**Fig. 4.**
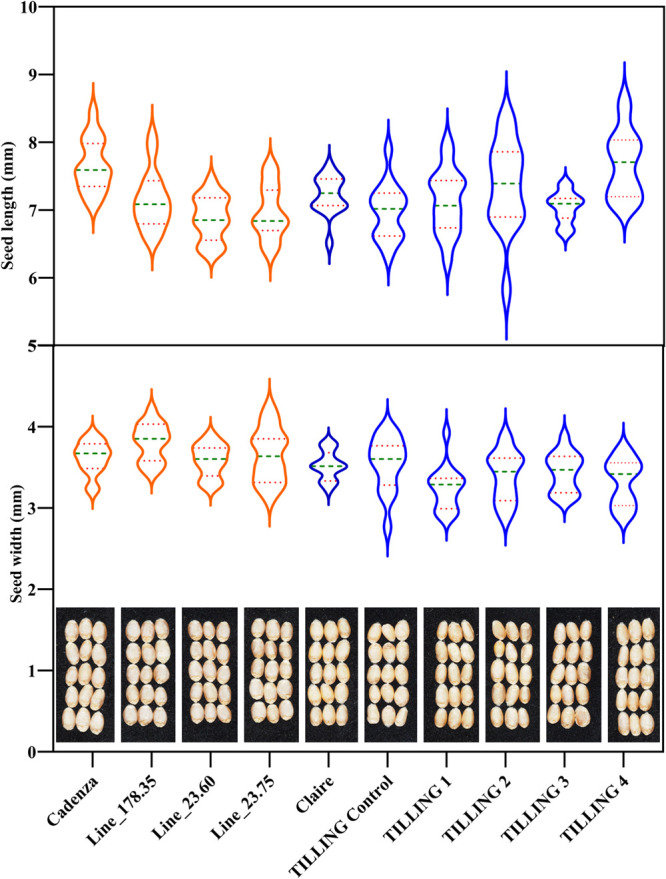
Violin plots illustrating the distribution of seed length (mm, upper panel) and seed width (mm, lower panel) across genome-edited lines (in orange) and TILLING mutants (in blue), and respective controls (Cadenza, Claire and TILLING control). Seed images are also shown for comparison. Green and dotted orange lines within the violin plots represent the median and upper/lower quartiles, respectively.

A key question, of course, was whether the knocking out of *TaASN2* affected the performance of the lines in the field. The study found no significant differences in grain yield between the edited lines and Cadenza (F_1,31.5_ = 0.95, *p* = 0.337) (Fig. 3C), although there were significant differences in thousand grain weight (TGW), with the edited lines showing a 10% reduction overall compared with Cadenza (F_1,35.8_ = 167.41, *p* < 0.001). This suggested that they produced more but smaller grains, and this is illustrated in Fig. 4, which shows the grains of Lines 23.60 and 23.75 to be shorter than those of the Cadenza control, with those of Line 178.35 of an intermediate length. Grain width was unaffected, and it is important to note that the grain weight and length were well within the normal range for wheat. It is possible that the perturbations in asparagine metabolism brought about by the editing of *TaASN2* affected seed set, seed number and resource allocation to each seed, but this requires further investigation. The grain of the edited lines was similar to the control with respect to nitrogen and carbon content, but did contain significantly more sulphur (F_1,46.0_ = 594.90, *p* < 0.001). That, too, requires further investigation, and is intriguing given the relationship between sulphur fertilisation and free asparagine accumulation.

In contrast to the GE lines, there were significant reductions in yield for the TILLING lines compared with Claire (F_1,29.9_ = 58.82, *p* < 0.001) (Fig. 3C), although changes in grain size were not so evident (Fig. 4). Chemical mutagenesis is a random process, of course, and the TILLING lines will contain thousands of mutations in addition to the ones identified in the *TaASN2* genes, even after backcrossing. The yield drag of the TILLING lines may reflect the effect of those ‘background’ mutations, making it a nice illustration of the advantage of using the targeted GE approach versus the random mutagenesis method. It should also be noted that the TILLING approach was only possible because of the relative simplicity of the gene family, with single *TaASN2* genes in each genome, and the assistance of a commercial plant breeder (RAGT, UK) in stacking the genes in the Claire background.

A second field trial was planted in early November 2022. A variation to the consent to release a GMO was sought and granted to enable the *TaASN1/TaASN2* double knockout line (Line 59) to be included for the first time. The trial also included *TaASN2* TILLING line total knockouts for the first time. Grain was harvested from this field trial in August 2023 and is currently being analysed.

## Field trials in a rapidly evolving regulatory scenario

EU regulations on GM and genome edited GE plants are enshrined in GM Food and Feed Regulation (EC) No. 1829/2003. The definition of a GMO used in this regulation was set out in Directive 2001/18/EC: a genetically modified organism (GMO) is an organism in which the genetic material has been altered in a way that does not occur naturally. Crop varieties carrying mutations induced by chemical or radiation treatment fall within this definition but do not have to go through the tortuous risk assessment process that is applied to transgenic plants before commercialisation in the EU (the Mutagenesis Exemption set out in Annex IB of Directive 2001/18/EC). In 2018, a European Court of Justice ruling on case C-528/16 meant that GE crops would not be granted a similar exemption. The ECJ judges were issuing a judgement on a case resulting from proceedings brought jointly by Confédération Paysanne, a French farmers’ union, and eight other organisations, against the French government. The plaintiffs sought an annulment of the exemption from GM regulations for organisms obtained by mutagenesis. The judges rejected annulment of the Mutagenesis Exemption, but stated that the exemption only applied to organisms obtained by methods of mutagenesis that have conventionally been used in a number of applications and have a long safety record, not organisms produced by genome editing. In coming to this judgement, the judges ignored the advice of the ECJ’s own Advocate General, and the scientific consensus on genome editing, and effectively put a full stop to the development of GE crops in the EU.

These regulations rolled over into UK law when the UK left the EU in 2020. However, the UK amended the regulations in March 2022 to include a different licensing process for the field release for research purposes of ‘qualifying higher plants’ in England. Qualifying higher plants were defined in that amendment as containing genetic changes that (a) could have occurred naturally; (b) could have been made using one or more of techniques such as polyploidy induction, mutagenesis and cell/protoplast fusion. This definition would include most GE plants as long as they did not contain a transgene. The approval process for field release of a qualifying higher plant is straightforward, with approval within 21 days. The UK’s Advisory Committee on Releases to the Environment (ACRE), an expert scientific committee that sits within the UK government’s Department for the Environment and Rural Affairs (DEFRA) and oversees the field release of GE and GM plants, has recently updated its guidance on qualifying higher plant research trials (ACRE 2023).

This was the first positive step in the regulation of crop biotechnology in the UK for a generation, and it was followed almost exactly a year later by completely new legislation in the form of the Genetic Technology (Precision Breeding) Act, which enables GE products to be brought to market. This act, which was granted royal assent on 23^rd^ March 2023, introduces the concept of a precision breeding organism (PBO). Decisions on whether or not a plant qualifies as a PBO will be made by ACRE. ACRE’s guidance on plants that will qualify as PBOs includes: genome edited plants with site-directed nuclease (SDN) Type 1 changes to the genetic material, made with non-homologous end joining (i.e., INDELS); SDN Type 2 and 3 changes, made with a DNA template and homologous end joining (i.e., allele replacement or insertion of a *cis*gene at a predetermined location); prime or base editing; epigenetic changes. It also includes other *cis*genic organisms. No transgenes must be present for a plant to qualify as a PBO, but plants produced by insertion of, for example, *Cas9*, gRNA and marker genes would qualify if those transgenes had been segregated away. On the other hand, plants with multiple simultaneous mutations resulting in a trait that is substantially different to one that might be reasonably expected to arise through natural processes or traditional methods may not qualify.

Although the Genetic Technology (Precision Breeding) Act has received royal assent, work is continuing to lay out the mechanisms by which the provisions of the Act will operate. One important aspect of that is the role of another scientific advisory committee, the Advisory Committee for Novel Foods and Processes, which sits within the UK’s Food Standards Agency (FSA).

At the time of writing, ACNFP’s position has not been finalised, but it is considering adopting a system where PBOs are classified as Tier 1 or Tier 2. Case by case risk assessment would only apply to Tier 2 products, which are broadly defined as PBOs or foods with compositional changes that could affect toxicity or allergenicity, or other potential safety concerns. The vast majority of PBOs would, therefore, be Tier 1, for which applicants would only be required to notify the FSA, describing the nature and purpose of the genetic change(s) that had been introduced. FSA would confirm that the PBO could be placed on the market for use in food and feed, and add the PBO to a public register (ACNFP 2023). Importantly, foods made from PBOs will not have to be labelled, as things stand.

These fast-moving regulatory developments are directly relevant to the low asparagine wheat project because we have now identified plants from Lines 23 and 59 that are transgene-free and, therefore, are qualifying higher plants/PBOs. Notification to the UKs Department of Environment and Rural Affairs of our intention to grow Line 59 plants in the field was published in June 2022 (DEFRA 2022).

## Future developments and concluding remarks

The acrylamide issue is becoming increasingly difficult for food manufacturers, with the prospect of Maximum Levels being introduced in 2024. Further big gains in reducing acrylamide formation in food from improvements in processing and quality control may be difficult to achieve, and food businesses will be looking to plant breeders and farmers to provide them with raw materials with reduced acrylamide-forming potential. We, therefore, urge wheat breeders to engage on the acrylamide issue and adopt low free asparagine concentration in the grain as a breeding target. One relatively straightforward action that breeders could take now would be to remove varieties/genotypes with a B genome *TaASN2* gene from their breeding programmes, but to our knowledge even that is not happening.

Beyond that, we have shown that large reductions in the free asparagine concentration of wheat grain are possible using genome editing. There was evidence of a small reduction in grain size in the low asparagine edited wheat in the first year of field trials, but there were no significant effects on yield or nitrogen content. Further field trials will have to be conducted to confirm this, but at present we have no evidence that the field performance of the wheat has been affected by the genetic interventions we have made.

The availability of low acrylamide wheat could enable food businesses to comply with evolving regulations on acrylamide without costly changes to production lines or reductions in product quality. It could also have a significant impact on dietary acrylamide intake for consumers. The availability of very low asparagine genotypes for incorporation into their breeding programmes may encourage wheat breeders to develop low asparagine commercial varieties. However, GE plants will only be developed for commercial use if the right regulatory framework is in place and breeders can be confident that they will get a return on their investment. We, therefore, welcome the legislation introduced by the UK government to enable the field release and commercialisation of qualifying higher plants and PBOs in England. We are also encouraged by the European Union’s declared intention to change its stance on GE crops as part of its ‘European Green Deal’ (European Commission 2023). This research area is an excellent example of how strategic and applied crop research is driven by regulations and also needs a supportive regulatory environment in which to thrive.

## Author Contribution Statement

NK prepared all the figures. NGH wrote the first draft of the manuscript, which was then edited and developed by all the authors.

## Figures and Tables

**Table 1.** The number of mismatches (out of 20) between the nucleotide sequences of the four gRNAs used to edit *TaASN2* and the four potential gRNA binding sites in *TaASN1*, *TaASN2*, *TaASN3.1*, *TaASN3.2* and *TaASN4*, and the presence/absence of a PAM sequence. The editing in *TaASN1* occurred at the target site for gRNA3. Note that the genes on the A, B and D genomes have identical nucleotide sequences at the target sites.

		gRNA1	gRNA2	gRNA3	gRNA4
*TaASN1*	Mismatches	7	3	2	8
	PAM intact?	Yes	Yes	Yes	No
*TaASN2*	Mismatches	0	0	0	0
	PAM intact?	Yes	Yes	Yes	Yes
*TaASN3.1*	Mismatches	5	7	5	12
	PAM intact?	No	No	No	Yes
*TaASN3.2*	Mismatches	7	5/6	4/5	12
	PAM intact?	No	No	No	No
*TaASN4*	Mismatches	5	5	3/4	10
	PAM intact?	No	No	No	No
